# Quantitative 2D Magnetorelaxometry Imaging of Magnetic Nanoparticles Using Optically Pumped Magnetometers

**DOI:** 10.3390/s20030753

**Published:** 2020-01-29

**Authors:** Aaron Jaufenthaler, Peter Schier, Thomas Middelmann, Maik Liebl, Frank Wiekhorst, Daniel Baumgarten

**Affiliations:** 1 Institute of Electrical and Biomedical Engineering, UMIT—Private University for Health Sciences, Medical Informatics and Technology, 6060 Hall in Tirol, Austria; 2 Department Biosignals, PTB—Physikalisch-Technische Bundesanstalt, 10587 Berlin, Germany; 3 Institute of Biomedical Engineering and Informatics, Technische Universität Ilmenau, 98693 Ilmenau, Germany

**Keywords:** magnetic nanoparticles, optically pumped magnetometers, magnetorelaxometry imaging

## Abstract

For biomagnetical applications exploiting physical properties of magnetic nanoparticles (MNP), e.g., magnetic hyperthermia, knowledge about the quantitative spatial MNP distribution is crucial, which can be extracted by magnetorelaxometry (MRX) imaging. In this paper, we present quantification, quantitative 1D reconstruction, and quantitative 2D imaging of MNP by exploiting optically pumped magnetometers for MRX. While highlighting the potential of commercially available optically pumped magnetometers (OPM) for MRXI, we discuss current limitations of the used OPM. We show, that with our OPM setup, MNP can be precisely quantified with iron amounts down to ≈6 μg, which can be improved easily. With a 1D-reconstruction setup, point-like and complex MNP phantoms can be reconstructed quantitatively with high precision and accuracy. We show that with our developed 2D MRX imaging setup, which measures 12 cm by 8 cm, point-like MNP distributions with clinically relevant iron concentrations can be reconstructed precisely and accurately. Our 2D setup has the potential to be easily extended to a tomography styled (and thus slice-selective) 3D scanner, by adding a mechanical axis to the phantom.

## 1. Introduction

Magnetic nanoparticles (MNP) offer a large variety of promising applications in medicine, e.g., magnetic hyperthermia and magnetic drug targeting [1]. For these applications, knowledge of the quantitative spatial MNP distribution is essential for treatment planning and supervision. This information can be gathered by means of magnetorelaxometry (MRX) imaging. In MRX, the magnetic moments of the superparamagnetic MNP are aligned by applying an external magnetic field, forming a net magnetic moment. After switching off the external field, the relaxation of the MNP’s net magnetic moment is monitored by sensitive magnetometers, e.g., superconducting quantum interference devices (SQUID) [2], fluxgates [3], and optically pumped magnetometers (OPM) [4]. The challenge in MRX is to operate magnetometers while withstanding magnetic field pulses in the mT range as well as acquiring the MNP relaxation signal with smallest possible dead times after shutting off the excitation field, at high bandwidth and with a high sensitivity. For slowly relaxing MNP, which are investigated here, also a small bandwidth may be sufficient for MRX experiments. By analyzing the relaxation curves, quantitative information about MNP can be extracted, e.g., saturation magnetization and anisotropy [5], iron concentration [6], and aggregation [7]. Additionally, the binding state [8,9] and size distribution [9,10] of the MNP can be reconstructed. In MRX imaging (MRXI), multiple MRX sequences with spatially different excitation fields and usually multiple sensors are performed [11,12]. By solving the ill-posed inverse problem, the quantitative spatial MNP distribution can be reconstructed.

The advantage of using OPM for MRX imaging would be a more flexible sensor positioning, which may be exploited to improve the ill-conditioned inverse problem of MRXI. OPM offer high sensitivities in the $fT/\sqrt{\mathrm{Hz}}$ range [13], while achieving small sensor-target distances of several mm. In contrast, SQUID need a dewar for thermal insulation, which usually limits the minimal sensor–target distance to several cm. In [11], a sensor-to-target distance of 45 mm is reported, while in [14] a minimal spacing of 12 mm was achieved. In the past, MRX experiments with OPM have been demonstrated by multiple groups, both with laboratory magnetometers [4,15], as well as with commercially available sensors [16]. In 2016, Dolgovskiy et al. [5] demonstrated magnetic source imaging with a custom built optically pumped magnetic field camera with a field of view of 20 mm by 20 mm. In this way, they could monitor magnetic field distributions. The inverse problem found in MRXI with multiple coils and/or sensors is usually ill-conditioned. In this paper, we present the development of a 2D MRX imaging setup using commercially available OPM and multiple excitation coils, and demonstrate measurements with MNP concentrations of clinical relevance. As prerequisite, we show the quantification of MNP samples with OPM. In contrast to other works on MRX with OPM [4,15,16], we apply nonhomogeneous excitation fields to the MNP, which offers improved imaging quality in MRXI [17].

## 2. Materials and Methods

### 2.1. Overview of Experimental OPM Setups for MRX Quantification and 2D Imaging

**Figure 1:**
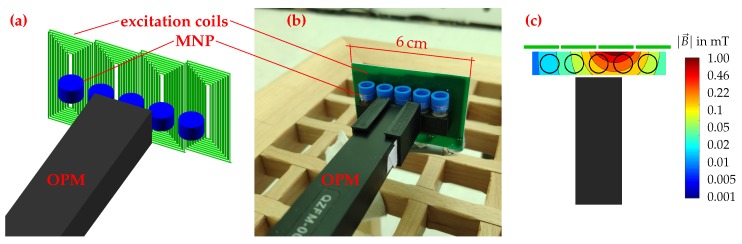
Setup for MNP quantification and quantitative 1D-reconstruction. Schematic representation (**a**) and photo (**b**). Simulated magnetic field map (**c**) for the third (from left to right) activated excitation coil. Please note the logarithmic scaling of the axis.

**Figure 2:**
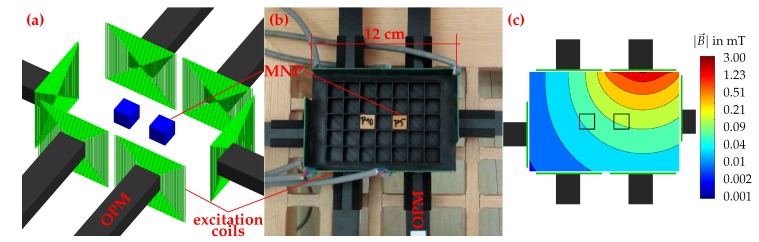
Setup for 2D MRX imaging. Schematic representation (**a**) and photo (**b**). Simulated magnetic field map (**c**) for the activated top-right excitation coil. Please note the logarithmic scaling of the axis.

The setup developed for MNP quantification and quantitative 1D-reconstruction is shown in Figure 1, while the setup developed for quantitative 2D-MRX imaging with OPM is shown in Figure 2. Both setups consist of several excitation coils for MNP alignment and one or six OPM, respectively. The MNP phantoms are positioned in 3D printed grids. All experiments were performed within the magnetically shielded room BMSR-2 at the PTB in Berlin [18].

### 2.2. MRX Excitation Coil Circuit

**Figure 3:**
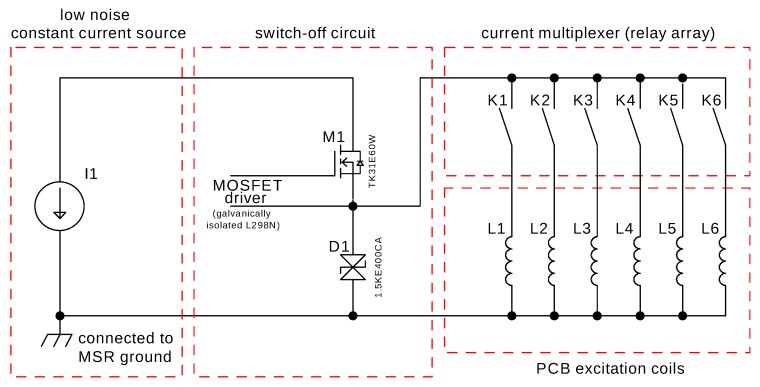
Simplified schematic of the coil driver: constant current source (I1), high voltage MOSFET (M1), TVS diode (D1), current multiplexer relays (K1–K6), and PCB excitation coils (L1–L6).

The excitation circuit used for MNP excitation consists of a current source, switch-off circuit, current multiplexer, and excitation coils. The circuit of the excitation system with a maximum current of about 1 A is sketched in Figure 3. The underlying principle of the excitation circuit is based on the design in [19]. The key for a fast and reproducible switch-off of the current, which is important to be able to detect the relaxation of the MNP as soon as possible after field removal, is a high-voltage field-effect transistor (M1) in combination with a high-voltage transient voltage suppression diode (D1). When shutting off (M1), the diode clamps the coil to a fixed maximum voltage, allowing for a fast decay of the magnetic field within several μs. The coil driver output is multiplexed by an array of relays, which is connected to the excitation coils. All coils are realized as double-layer printed circuit boards. In the quantification and 1D-reconstruction experiments, each of the 18-turn excitation coils has a dimension of 15 mm by 35 mm, whereas the 54-turn coils in the 2D imaging experiment have dimensions of 53 mm by 53 mm. Each coil has an inductance of <100 μH.

### 2.3. OPM

The OPM used in this work are commercially available QZFM magnetometers from QuSpin (QuSpin Inc., Louisville, CO, USA), provided by the PTB’s core faciltiy Metrology of Ultra-Low Magnetic Fields. The magnetometers operate in the spin exchange relaxation-free (SERF) regime [13,20]. As a result, the OPM can only operate at very low background magnetic fields (or they have to be compensated) and have a dynamic range of ±5 nT. The bandwidth is specified as 135 Hz, with a sensitivity of approximately $15\;fT/\sqrt{\mathrm{Hz}}$ [21]. The gas cell has a volume of 27 mm^3^ and the center of the sensitive volume is located 6 mm behind the sensor front.

### 2.4. Setup and Procedure for MNP Quantification and 1D Reconstruction

The setup for MNP quantification and 1D reconstruction (Figure 1a,b) consists of a single OPM, four excitation coils, and a 3D printed sample holder for up to five vials, filled with MNP. For these experiments, a dilution series of immobilized Resovist^®^ (Schering, Berlin, Germany) MNP was prepared. The MNP with a hydrodynamic diameter of 45 nm show a bimodal core size distribution with peaks at 5 nm and 24 nm [22]. The eight MNP samples with a sample volume of 140 μL and an iron amount ranging from 139.2 μg (≈1 mg/cm^3^ iron concentration) down to 5.8 μg (≈41 μg/cm^3^ iron concentration) were freeze dried in Mannitol.

For the MNP quantification experiment, a single MNP sample is positioned directly in front of the OPM. Then, the third coil (from left to right, Figure 1) is pulsed for one second, producing an inhomogeneous field of <1mT at the MNP location. The simulated magnetic field map of the excitation field is shown in Figure 1c. After switching-off the external field, the relaxation is recorded and the relaxation amplitude extracted (see Section 2.7). This process is repeated for all samples in the dilution series.

For the 1D reconstruction experiment, an MNP pattern composed of samples from the dilution series was inserted into the 3D printed sample holder. Then, the four excitation coils were pulsed consecutively for one second each, while in between the relaxation signal was recorded for 10 s. This procedure was repeated for different MNP patterns.

### 2.5. Setup and Procedure for 2D Imaging

The setup for 2D imaging (Figure 2a,b) consists of six OPM, six excitation coils, and a 3D printed 12 cm by 8 cm sample holder for MNP cubes. The excitation coils and OPM are positioned on four sides of the sample holder.

For this experiment, Berlin Heart MNP (Berlin Heart GmbH, Berlin, Germany) with a mean core diameter of 20 nm were immobilized in gypsum cubes with edge length of 12 mm. Each cube was loaded with an iron concentration of 3.70 mg/cm^3^ [8].

For the 2D reconstruction experiment, an MNP pattern composed of MNP enriched gypsum cubes was inserted into the 3D printed sample holder. Then, the excitation coils were pulsed consecutively for one second each, while in between the relaxation signal was recorded for 10 s. The simulated magnetic field map of a single excitation field is shown in Figure 2c. After the extraction of the relaxation amplitudes for each OPM for each excitation pulse (see Section 2.7), the mathematical inverse problem was solved for the 2D quantitative spatial MNP concentration (see Section 2.8). This procedure was repeated for different MNP patterns.

### 2.6. MRX Model

The relaxation dynamic of MNP’s magnetic moments can be described by two parallel occurring processes, namely Néel relaxation and Brownian relaxation. Although in Brownian relaxation the magnetic moment rotates due to mechanical motion of the whole nanoparticle, in Néel relaxation, only the magnetic moment flips, while the particle remains stationary. The Brownian relaxation time [23] depends on the viscosity τ, the particle’s hydrodynamic volume $V_{h}$, and the temperature *T*:

(1) $$\tau_{B}=\frac{3\tau V_{h}}{k_{B}T}.$$

By immobilizing the MNP, which was done for all samples in our experiments, Brownian relaxation can be suppressed [24]. The zero field Néel relaxation time constant is defined as [25]

(2) $$\tau_{N}=\tau_{0}\exp\left(\frac{KV_{c}}{k_{B}T}\right),$$

where the damping time is $\tau_{0}$, the effective magnetic anisotropy is *K*, the particle’s core volume is $V_{c}$ the Boltzmann constant $k_{B}$, and the temperature *T*. The decay of the MNP’s net magnetic moment leads to a time dependent magnetic flux density B(t) at the sensor location. Assuming an ensemble of identical MNP, the relaxation can be described as

(3) $$B\left(t\right)=B_{0}\;\cdot \;\exp\left(-\frac{t}{\tau_{N}}\right)+B_{\mathrm{offset}},$$

with the relaxation amplitude $B_{0}$ and time after the start of the relaxation *t*. $B_{\mathrm{offset}}$ is introduced to model remanent magnetizations of MNP and the environment. In real MNP samples, the diameter distribution is nonuniform. Here, the relaxation can be described phenomenologically by a stretched exponential [26]

(4) $$B\left(t\right)=B_{0}\;\cdot \;\exp\left[-{\left(\frac{t}{\tau_{N}}\right)}^{\gamma }\right]+B_{\mathrm{offset}},$$

with the stretching parameter γ.

Given the magnetometer bandwidth of 135 Hz, the size range of MNP contributing to the MRX signal can be estimated. When considering Néel relaxation only, which is appropriate for immobilized particles, (2) may be used to calculate the detectable diameters. $\tau_{0}$ is usually in the range of $10^{-8}\;s$ to $10^{-12}\;s$ [14]. We assume a spherical magnetite particle with a typical anisotropy of $10^{4}\;J/m^{3}$, T=290 K and detectable time constants of 1/135 s to 60 s. With $\tau_{0}=10^{-9}\;s$, the calculated MNP core diameters range from 23 nm to 27 nm. If Néel relaxation and Brownian relaxation occur in parallel, an effective time constant can be formulated as [27]

(5) $$\tau_{\mathrm{eff}}=\frac{\tau_{N}\;\cdot \;\tau_{B}}{\tau_{N}+\tau_{B}}.$$

When evaluating $\tau_{\mathrm{eff}}$ for different (hydrodynamic and core) diameters, it comes out that MNP with a core diameter larger than 23 nm, while having a hydrodynamic diameter larger than 300 nm, can be detected with a 135 Hz bandwidth. Similar calculations for SQUID-MRX can be found in [14].

### 2.7. Data Acquisition and Preprocessing

The raw OPM data was acquired by digitizing the analog output, while the coil current was recorded synchronously. The data was recorded with a sample rate of 1 kHz, as the OPM has a bandwidth of 135 Hz.

The acquired data for each excitation pulse was time-aligned using the coil current slope and averaged five times. The data was then fitted to the stretched exponential relaxation model (4) using the Trust-Region-Reflective Least Squares algorithm [28] provided by Matlab^®^ (R2017b).

### 2.8. System Model and Reconstruction

For quantitative 1D reconstruction and 2D imaging, a forward model for MRX is necessary. Thus, the physical, geometrical, and magnetic properties of the MRX system are modeled mathematically by the system of linear equations

(6) $$L\;\cdot \;\vec{c}=\vec{b},$$

where the lead field matrix $L\in R^{N_{s}N_{a}\times N_{v}}$ links the MNP concentration of each voxel, denoted by the vector $\vec{c}\in R^{N_{v}}$, to the measured relaxation amplitudes $\vec{b}\in R^{N_{s}N_{a}}$. In our 1D reconstruction experiment, $N_{s}=1$, $N_{a}=4$, and $N_{v}=5$, while in our 2D imaging experiment $N_{s}=6$, $N_{a}=6$, and $N_{v}=35$. Each of the $N_{a}$ different externally applied magnetic fields can be expressed as an $N_{s}\times N_{v}$ sized matrix that contains the impact of the $N_{v}$ voxels on all $N_{s}$ sensors. These $N_{a}$ individual measurements are concatenated below each other to construct the final lead field matrix L and the complete measurement vector $\vec{b}$. The component $l_{s,v}$ in the sth row and the vth column of the lead field matrix referring to the ath magnetic field application is given by

(7) $$l_{s,v}=\frac{\mu_{0}\chi \Delta \kappa_{1,2}}{4\pi }\left(\frac{3{\vec{n}}_{s}^{T}\left(\left({\vec{r}}_{s}-{\vec{r}}_{v}\right){\left({\vec{r}}_{s}-{\vec{r}}_{v}\right)}^{T}\right)}{{\left\|{\vec{r}}_{s}-{\vec{r}}_{v}\right\|}^{5}}-\frac{{\vec{n}}_{s}^{T}}{{\left\|{\vec{r}}_{s}-{\vec{r}}_{v}\right\|}^{3}}\right){\vec{h}}_{a,v},$$

where $\mu_{0}=4\pi \;\cdot \;10^{-7}\;H/m$ denotes the magnetic constant, χ the magnetic susceptibility of the MNP, and $\Delta \kappa_{1,2}$ is a constant defined by the relaxation properties. The location of voxel *v* is given by the vector ${\vec{r}}_{v}$. The OPM are modeled as vectorial magnetometers with point-like sensitivity measured at position ${\vec{r}}_{s}$ in the direction ${\vec{n}}_{s}$. The magnetic field strength of the $a^{\mathrm{th}}$ magnetic field application in voxel *v* is denoted by the vector ${\vec{h}}_{a,v}$. For a deeper understanding of the construction of the lead field matrix, the reader is referred to the work in [8]. Usually, each voxel is modeled as point-like source of a decaying magnetic moment at location ${\vec{r}}_{v}$. This introduces model inaccuracies in the system equations, in particular for coarse voxel grids with small coil and sensor distances to the voxels. Therefore, each voxel is subdivided into 11×11×11 subvoxels. The respective lead field matrix components of the subvoxels are calculated individually with (7) and are averaged subsequently to get refined measures for the voxel-sensor interactions $l_{s,v}$, increasing the model fidelity.

The system matrix of an MRX forward model is typically ill-conditioned. It is widely known that the condition number of a matrix $\mathrm{cond}\left(L\right)=∥L∥∥L^{+}∥$ (with $L^{+}$ being the pseudoinverse of L) is an indicator on how strongly measurement noise is amplified when solving the inverse problem. This characteristic is also evident from the inequality

(8) $$\begin{array}{c} \frac{∥\delta x∥}{∥x∥}\leq \mathrm{cond}\left(L\right)\;\cdot \;\frac{∥\delta b∥}{∥b∥}, \end{array}$$

with δb denoting the measurement noise and δx the deviation from the true solution x [29]. Therefore, it is important to choose a proper regularization method that incorporates a priori knowledge about the solution in order to minimize the impact of the ill-conditioning.

The reconstructions of the MNP distributions are realized by solving the inverse problem to (6) using an iterative Tikhonov regularization [30] with added non-negativity constraint. The corresponding optimization problem can be stated in the form

(9) $$\begin{array}{cc} \tilde{\vec{c}}= & \underset{\vec{c}}{\arg\min}\;{\left\|L\vec{c}-\vec{b}\right\|}^{2}+\alpha {\left\|I\vec{c}\right\|}^{2}\\ & s.\;t.\;\vec{c}⪰0 \end{array}$$

with α denoting the regularization parameter and $I\in R^{N_{v}\times N_{v}}$ a weighting matrix which is chosen as identity matrix here. The gradient $\vec{g}\left(\vec{c}\right)$ of the objective function in (9) is given by

(10) $$\begin{array}{c} \vec{g}\left(\vec{c}\right)=\left(L^{T}L+\alpha I\right)\vec{c}-L^{T}\vec{b} \end{array}$$

and can be used to formulate the final non-negative, iterative Tikhonov regularization scheme

(11) $$\begin{array}{c} {\vec{c}}_{j+1}=\Pi_{R_{0}^{+}}\left({\vec{c}}_{j}-\beta \vec{g}\left({\vec{c}}_{j}\right)\right), \end{array}$$

where $\Pi_{R_{0}^{+}}$ denotes the non-negativity enforcing projection operation $\Pi_{R_{0}^{+}}\left(\vec{c}\right)=\max\left\{0,\;\vec{c}\right\}$ for every vector component and β defines the step size for the gradient descent.

Pearson’s rank correlation coefficient is used as a measure for reconstruction precision.

## 3. Results

### 3.1. OPM

**Figure 4:**
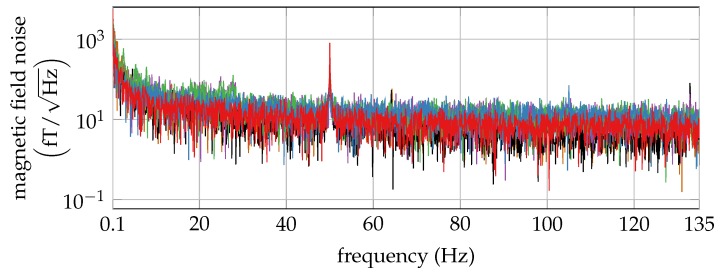
Noise amplitudes for each of the six optically pumped magnetometers (OPM).

Figure 4 shows the noise amplitude of one OPM inside the BMSR-2. The noise floor was about 15 fT/Hz, dominated by sensor noise. Multiple OPM did not interfer in terms of noise, as the distance between them was large enough (≥4 cm).

The OPM dead time after switching-off the external magnetic field was estimated based on measurements by applying a small oscillating field to the OPM during and after the magnetization pulse. The oscillating field recovered 20 ms after switching-off the excitation pulse.

### 3.2. MNP Quantification

**Figure 5:**
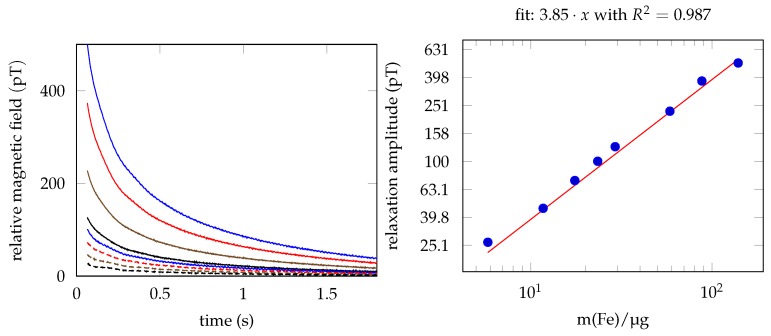
(**a**) Raw relaxation data, measured with a single OPM for the dilution series. The data within the sensor dead time is not shown. (**b**) Relaxation amplitude fits with linear regression of iron concentration vs. relaxation amplitude.

The relative (offset subtracted), unfiltered relaxation signals of each sample from the dilution series is shown in Figure 5a. The fitted relaxation amplitudes are shown in Figure 5b. Each fit matches with an adjusted $R^{2}$ of >0.99. The linear fit of the relaxation amplitude vs. the iron concentration shows a $R^{2}$ of 0.987.

### 3.3. 1D Reconstruction

**Figure 6:**
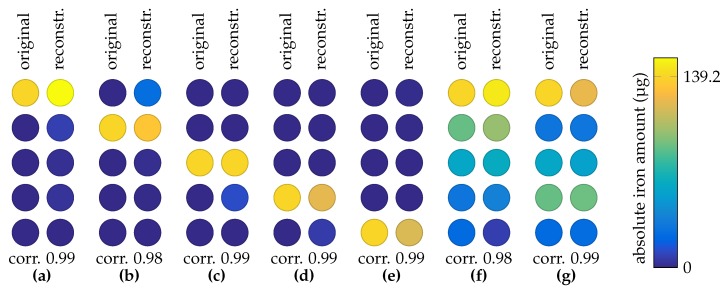
1D reconstruction: ground truth and reconstruction for each magnetic nanoparticle (MNP) phantom. Point-like MNP phantoms in (**a**–**e**) and dilution series phantoms in (**f**,**g**).

An MNP phantom was placed in the 3D printed sample holder, the coils were pulsed and the relaxation signals were recorded with the OPM. As described in Section 2.8, the four sequential activations lead to four relaxation amplitudes, after fitting the relaxation model (4). With these four amplitudes and the mathematical model of the system, the concentration at each of the five positions in the 3D printed sample holder is reconstructed. It should be noted that the condition number of this setup is low (<10), indicating that the inverse problem is well conditioned. The ground truth and the reconstructed MNP distribution for various phantom configurations are shown in Figure 6. The regularization parameter α was set beforehand and left fixed for all phantoms. First, a point-like MNP phantom (139.2 μg iron amount) was placed one end of the sample grid (Figure 6a) and then shifted to the other end of the grid (Figure 6b–e). It can be seen that point-like phantoms are reconstructed with high spatial precision and quantitative accuracy. Then a dilution series composed of five MNP vials in a descending iron concentration order is placed in the 3D printed sample holder. The well reconstructed phantom is depicted in Figure 6f. Finally, the order of the samples was shuffled and then the distribution was reconstructed (Figure 6g), still with a high correlation.

### 3.4. 2D MRX Imaging

**Figure 7:**
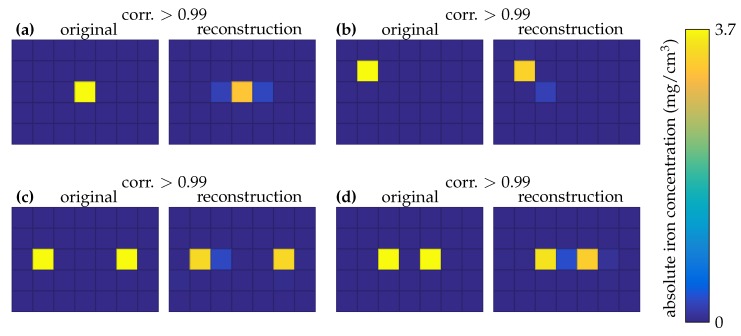
2D imaging: ground truth and reconstruction for each magnetic nanoparticle phantom.

**Figure 8:**
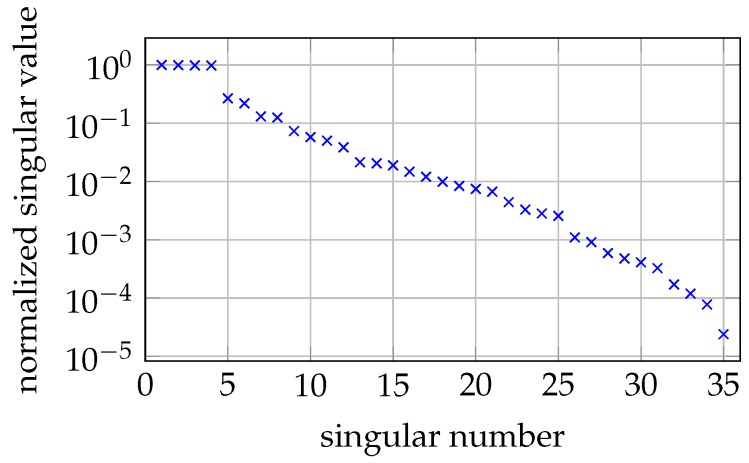
Singular values of the inverse problem of our 2D imaging setup.

Various MNP phantoms were placed, one at a time, in our 2D OPM MRXI setup. The reconstruction results are depicted in Figure 7. Note that the regularization parameter α was set once and left fixed for all of the reconstructions. The inverse problem is ill-conditioned with a condition number of $\approx 4\times 10^{4}$. The singular values are depicted in Figure 8.

To characterize the setup, the MNP phantoms were selected carefully. First, a point-like source was placed at the center of the sample grid (Figure 7a) and then moved near the corner of the field of view (Figure 7b). To characterize the setup for selectivity, two MNP cubes are placed at a center-to-center distance of ≈5.5 cm and then approached to a center-to-center distance of ≈3 cm (Figure 7c,d).

## 4. Discussion

When switching-off our excitation coil, we determined the OPM dead time in the range of 20 ms. A similar dead time was measured in [16]. Combined with the bandwidth of 135 Hz, the OPM is well suited for MRX of slowly relaxing MNP, but only of limited use for fast relaxing particle systems. It has been calculated that in our experiments with immobilized MNP, only particles with a core diameter of ≈23 nm to ≈27 nm contribute to the measured MRX signal. Although the calculations are straight forward, note that these values should be considered as rough estimates, since parameters like the anisotropy constant *K* and the damping time $\tau_{0}$ have a large impact and are not known precisely. For a detailed investigation of $\tau_{0}$, the reader is referred to [31]. Besides, the magnetometer dead time, which leads to an increase of the lower limit of the detectable diameter range, was not included in our estimations.

The quantification of MNP with our setup was demonstrated for amounts down to 5.8 μg of iron. Although this might sound reasonably sensitive, note that the MNP samples were placed very close to the coil and the OPM, which usually is not the case in MRXI. The detection limit of the current setup might be increased, e.g., by increasing the excitation coil current. We presume, that the amplitude deviations from the theoretical linear response relation arise mainly from positioning inaccuracy of the MNP samples. Additionally, for the quantification it is noted, that based on the coil geometry, a magnetic gradient is applied to the MNP, causing an inhomogeneous excitation. However, as all the samples from the dilution series are placed at the same position, the gradient affects all samples in an equal manner, and thus can be neglected here. However, the gradient has to be taken into account to calculate the MNP’s property $\chi \Delta \kappa_{1,2}$. This is required as scaling factor for MRX imaging. In 1D reconstruction and 2D imaging experiments, the gradient produced by the coils is included in the inverse problem (see Section 2.8). For our 2D imaging experiments, the iron concentration was selected to be of clinical relevance [24]. We would like to emphasize our motivation for sensor and coil placement in the 2D MRXI setup. Although the inverse problem of (2D) MRXI would greatly benefit from a placement of coils and/or sensors on the top and bottom of the sample holder, the aim for this setup is to allow for a tomography-styled (and thus slice selective) MNP scanner. Therefore, the components can only be placed around the phantom.

## 5. Conclusion and Outlook

In this paper, we presented quantification, 1D reconstruction, and 2D imaging of MNP by employing commercially available OPM for MRXI. Although the principal applicability of OPM for MRX has been shown before, only homogeneous excitation fields were used [4,15,16]. This would limit MRXI, as the performance can be increased significantly by applying inhomogeneous fields [17]. We have shown that immobilized MNP can be quantified accurately with OPM, even when applying gradients. Further, it was possible to extend the setup to 1D reconstruction of MNP distributions, by the utilization of multiple excitation coils. Finally, we have shown the development of a 2D OPM MRX imaging setup. With this setup, we demonstrated the use of multiple OPM for MRXI with a 12 cm by 8 cm field of view. Point-like MNP distributions with iron concentrations of clinical relevance could be reconstructed precisely. In the future, we will work on improving the spatial sensitivity of our 2D imaging setup and to extend it to a 3D imaging (scanner). To achieve that, the coil geometries and coil currents, as well as the coil and sensor positions will be optimized with respect to the condition number of the lead field matrix L [32,33]. Due to the simple composition of the imaging setup, it may also be adopted to operate in a small sized magnetic shielding, leading to a portable MRX imaging system. Here, care has to be taken about the static and dynamic magnetization of the magnetic shield.

## Abbreviations

The following abbreviations are used in this manuscript:

MOSFET	Metal Oxide Semiconductor Field-Effect Transistor
MNP	Magnetic nanoparticle
MRX	Magnetorelaxometry
MRXI	Magnetorelaxometry imaging
OPM	Optically pumped magnetometer
PCB	Printed circuit board
SERF	Spin-exchange-relaxation-free
SQUID	Superconducting quantum interference device
